# Detection of prion seeding activity in the olfactory mucosa of patients with Fatal Familial Insomnia

**DOI:** 10.1038/srep46269

**Published:** 2017-04-07

**Authors:** Veronica Redaelli, Edoardo Bistaffa, Gianluigi Zanusso, Giulia Salzano, Luca Sacchetto, Martina Rossi, Chiara Maria Giulia De Luca, Michele Di Bari, Sara Maria Portaleone, Umberto Agrimi, Giuseppe Legname, Ignazio Roiter, Gianluigi Forloni, Fabrizio Tagliavini, Fabio Moda

**Affiliations:** 1 IRCCS Foundation Carlo Besta Neurological Institute, Department of Neurology 5 and Neuropathology, Milan, Italy; 2 Scuola Internazionale Superiore di Studi Avanzati (SISSA), Department of Neuroscience, Trieste, Italy; 3 University of Verona, Department of Neurosciences, Biomedicine and Movement Sciences, Verona, Italy; 4 University of Verona, Otolaryngology department, Verona, Italy; 5 Istituto Superiore di Sanità, Department of Veterinary Public Health and Food Safety, Rome, Italy; 6 Otolaryngology Unit, San Paolo Hospital, Department of Health Sciences, University of Milan, Milan, Italy; 7 ELETTRA Laboratory, Sincrotrone Trieste S.C.p.A., Trieste, Italy; 8 ASL9 Ca’Foncello, Treviso, Italy; 9 IRCCS Foundation Istituto di Ricerche Farmacologiche Mario Negri, Department of Neuroscience, Milan, Italy

## Abstract

Fatal Familial Insomnia (FFI) is a genetic prion disease caused by a point mutation in the prion protein gene (*PRNP*) characterized by prominent thalamic atrophy, diffuse astrogliosis and moderate deposition of PrP^Sc^ in the brain. Here, for the first time, we demonstrate that the olfactory mucosa (OM) of patients with FFI contains trace amount of PrP^Sc^ detectable by PMCA and RT-QuIC. Quantitative PMCA analysis estimated a PrP^Sc^ concentration of about 1 × 10^−14^ g/ml. In contrast, PrP^Sc^ was not detected in OM samples from healthy controls and patients affected by other neurodegenerative disorders, including Alzheimer’s disease, Parkinson’s disease and frontotemporal dementia. These results indicate that the detection limit of these assays is in the order of a single PrP^Sc^ oligomer/molecule with a specificity of 100%.

Transmissible Spongiform Encephalopathies (TSEs) are a group of fatal and incurable neurodegenerative diseases that might present as sporadic, acquired or genetic conditions. The most common form of sporadic TSE is represented by the Creutzfeldt-Jakob disease (CJD)^1^,^2^. Acquired forms include variant CJD (vCJD) which is linked to the consumption of cattle infected with bovine spongiform encephalopathy (BSE)^3^,^4^. Genetic forms are associated to mutations and insertions in the *PRNP* gene encoding the cellular prion protein (PrP^C^)^5^. Fatal Familial Insomnia (FFI) is caused by a point mutation at codon 178 of *PRNP* resulting in aspartic acid to asparagine substitution in coupling phase with methionine at polymorphic position 129 (D178N/129M)^6^. FFI is associated with sleep alterations, autonomic dysfunction, loss of weight and motor signs^7^. After symptoms onset, the disease usually leads to death within 2 years and no treatments are available at present^8^.

Although characterized by a great heterogeneity of clinical and neuropathological phenotypes, these diseases share a common infectious agent which is a conformationally altered form of the PrP^C^, known as PrP^Sc^, which is able to induce the conformational conversion of PrP^C^ into new PrP^Sc^. Compared to PrP^C^, PrP^Sc^ has higher content in β-sheet structures, is insoluble in mild detergents and partially resistant to protease degradation. Thus, after digestion with proteinase K (PK), PrP^C^ is completely digested while PrP^Sc^ shows a PK resistant core (27–30 kDa)^9^. Mutations in the *PRNP* gene might favor the process of PrP^C^ misfolding and aggregation thus favoring the formation of PrP^Sc^.

Both PrP^C^ and PrP^Sc^ can be variably glycosylated and give rise to diglycosylated, monoglycosylated and unglycosylated species^10^. After PK digestion, only the unglycosylated band can migrate at around 21 kDa (type 1) or 19 kDa (type 2)^1^. The relative abundance of each PrP glycoform (glycoform ratio) and the electrophoretic mobility of the unglycosylated form enable a biochemical classification of different prion strains. For instance, the sporadic forms of CJD are generally characterized by a predominance of the monoglycosylated PrP band, with unglycosylated PrP migrating at 21 kDa (sCJD-type1) or 19 kDa (sCJD-type2). BSE, vCJD and FFI are characterized by an abundance of the diglycosylated band with the unglycosylated one migrating at 19 kDa (type 2)^3^,^8^,^11^.

Recently, two innovative assays named Protein Misfolding Cyclic Amplification (PMCA) and Real Time Quaking-Induced Conversion (RT-QuIC) were generated to model the process of prion misfolding *in vitro* in an accelerated manner. PMCA^12^ consists of cycles of incubation and sonication of samples containing small amount of PrP^Sc^ in the presence of an excess of PrP^C^. This enables the exponential amplification of PrP^Sc^, and can begin the reaction with the equivalent of a single molecule of PrP^Sc^, which after amplification can give rise to billions of PrP^Sc^ molecules^13^. Therefore, PMCA analysis allowed to detect extremely low levels of PrP^Sc^ in urine^14^ and blood^15^,^16^ of patients with vCJD as well as in blood of sheep and primates experimentally infected with vCJD in preclinical stages^17^. RT-QuIC^18^ alternates phases of incubation to phases of vigorous shaking where soluble recombinant prion proteins (recPrP) are used as substrate for detecting femtomolar amounts of PrP^Sc^. The reaction is monitored by Thioflavin-T (ThT) fluorescence and enabled detection of PrP seeds in the cerebrospinal fluid (CSF) of patients with sporadic CJD (sCJD) or genetic forms of prion disease (e.g. GSS and FFI) either in the symptomatic^19^ or pre-symptomatic stage of the disease^20^,^21^. RT-QuIC has been further adapted to detect PrP^Sc^ in other tissues, such as the olfactory mucosa (OM) of patients suffering from sporadic CJD^22^. Taken together, these findings suggest that RT-QuIC and PMCA have a huge potential to detect trace-amount of PrP^Sc^ (≥1 femtogram) in peripheral tissues.

By taking advantage of recent observations^23^,^24^,^25^, we have optimized PMCA and RT-QuIC and extended their use for the analysis of OM samples collected from two patients with FFI, 16 patients with other neurodegenerative disorders including Alzheimer’s disease (AD), Parkinson’s disease (PD), Frontotemporal Dementia (FTD) and 10 healthy control (HC). Our results showed that both techniques were able to detect PrP^Sc^ in FFI-OM samples in the order of a single molecule with 100% specificity.

## Results

### PMCA and RT-QuIC efficiently detect PrP^Sc^ in serial dilutions of FFI brain homogenates

**Figure 1:**
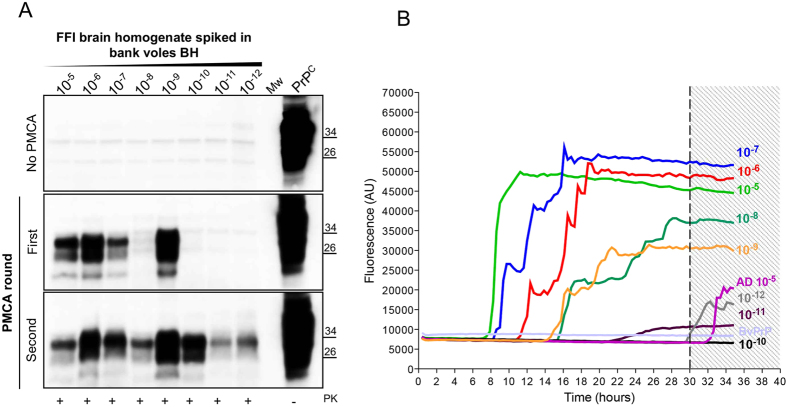
Serial dilutions of FFI brain homogenate (D178N/129M) were analyzed by means of PMCA and RT-QuIC for estimating their ability to detect PrP^Sc^. (**A**) Ten % bank vole brain homogenates (BvBH) were spiked with serial dilution of FFI brain homogenate (from 10^−5^ to 10^−12^). The signal of PrP^Sc^ was assessed by means of Western blotting, after PK digestion, with the 6D11 antibody. Mw refers to the molecular weight. PrP^C^ refers to normal bank vole brain homogenate not digested with PK. (**B**) Serial dilutions of FFI brain homogenate were analyzed by means of RT-QuIC reactions using BvPrP (90-231) as substrate. Average ThT fluorescence were plotted against time. AD brain homogenate (dilution 10^−5^) was used as negative control. BvPrP refers to unseeded recombinant protein.

To assess whether PrP^Sc^ associated to FFI (FFI-PrP^Sc^) was able to amplify by means of PMCA, we have performed spiking experiment diluting FFI brain homogenate (from 10^−5^ to 10^−12^ dilution of the brain) into 10% healthy bank vole brain homogenates (BvBH) carrying methionine at position 109 of the prion protein (M109). After one round of amplification, we could efficiently detect up to a 10^−9^ dilution while, after two rounds, we were able to amplify all dilutions (Fig. 1A). Similarly, dilutions of FFI brain homogenate were prepared in PBS and analyzed by means of RT-QuIC. The experiments were carried out using recombinant truncated form of the bank vole PrP (BvPrP(90-231)) as substrate and were seeded in triplicate with 2 μL of each dilution (Fig. 1B). All reactions were performed at least three times by different operators. We could detect up to 10^−9^ dilution of PrP^Sc^, while we did not efficiently detect any signal from higher dilutions. In particular, lower dilutions of brain homogenate induced a faster aggregation of BvPrP(90-231) (at around 8 hours), while intermediate dilutions induced aggregation after 14 hours. A sample was considered positive when the mean of the highest two fluorescence values (AU) of the replicates was higher than 10.000 AU and at least two out of three replicates crossed this value before 30 hours (*see materials and methods for details*). Samples that did not cross the threshold before 30 hours were considered negative. Brain homogenates of patient with AD and FTD were used as control and induced aggregation of BvPrP(90-231) after 30 hours, thus were considered negative (Fig. 1B).

### PMCA and RT-QuIC can detect PrP^Sc^ from samples of olfactory mucosa

**Figure 2:**
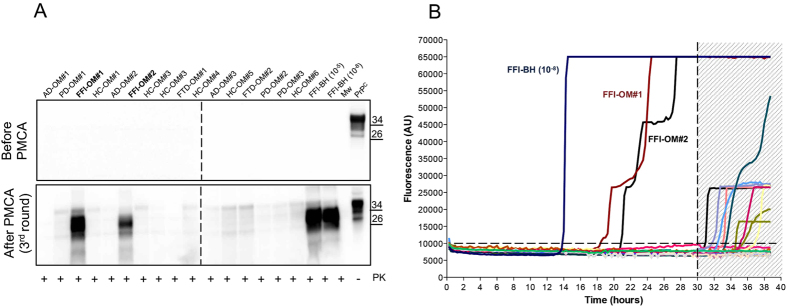
PrP^Sc^ detection in OM of FFI subjects using PMCA and RT-QuIC assays. (**A**) Representative image of PMCA analysis of olfactory mucosa samples (n = 28) that were blindly performed. After three rounds of amplification PrP^Sc^ could be detected in two samples (FFI-OM#1 and FFI-OM#2) that belong to subjects that were symptomatic at the time of OM collection. 10^−5^ and 10^−8^ refer to dilutions of FFI brain homogenate in PMCA substrate that were used as positive control for reaction. The signal of PrP^Sc^ was assessed by means of Western blotting, after PK digestion, with the 6D11 antibody. Numbers in the right indicate the position of Mw. PrP^C^ refers to normal bank vole brain homogenate that was not digested with PK. Dashed line indicates cropped images from separate gels. (**B**) RT-QuIC reactions were seeded with olfactory mucosa specimens of all patients. FFI brain homogenate (FFI-BH 10^−8^) was used as positive control for the reaction. Two OM samples were found positive before the threshold set at 30 hours. Average ThT fluorescence were plotted against time.

**Figure 3:**
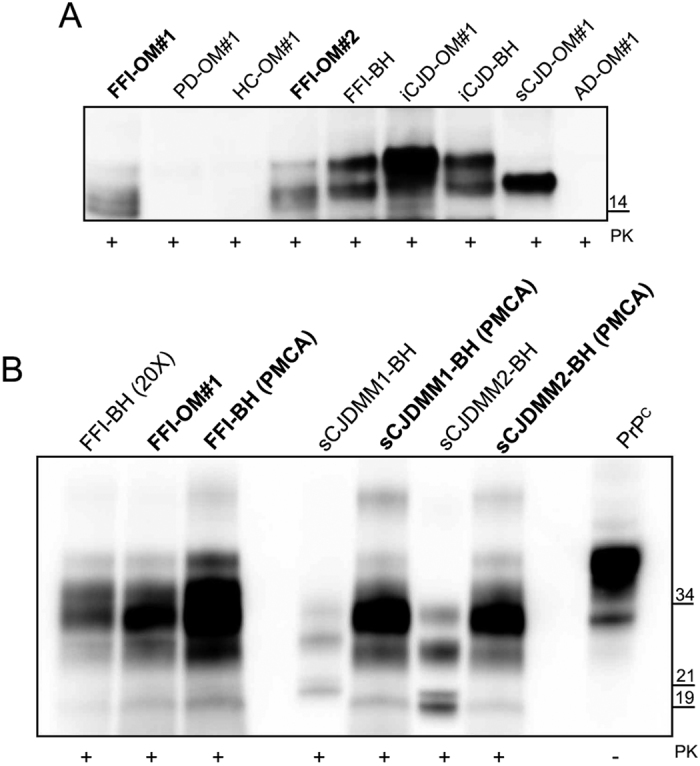
Amplified OM-PrP^Sc^ comparison with that of FFI or sCJD-type 1 and sCJD-type 2 brain homogenates. (**A**) Western blot of the final products of RT-QuIC reaction seeded with brain homogenates or olfactory mucosa from sCJD, iCJD, FFI, AD, PD patients and healthy controls (HC). Samples were digested with PK [50 μg/mL] and immunoblot was probed with the C-terminal antibody SAF-84. Number in the right indicates the position of Mw. (**B**) Biochemical profile of amplified products (both brain homogenates and olfactory mucosa) was compared to that of FFI, sCJD-type 1 and sCJD-type 2 brain homogenates assessed before and after PMCA. FFI brain homogenate was concentrated 20-fold for PrP^Sc^ detection. The signal of PrP^Sc^ was assessed by means of Western blotting, after PK digestion, with the 6D11 antibody. Numbers in the right indicate the position of Mw: 21 kDa refers to type 1 PrP^Sc^, while 19 kDa refers to type 2 PrP^Sc^.

OM samples (n = 28) collected from patients with FFI (n = 2), AD (n = 6), PD (n = 6), FTD (n = 4) and HC (n = 10) were blindly analyzed by means of PMCA and RT-QuIC. After three rounds of PMCA, we could detect PrP^Sc^ only in FFI-OM samples (Fig. 2A). All the other samples remained negative even after 7 rounds of amplification (Supplementary Fig. 1). Similarly, through RT-QuIC analysis we could detect prion seeding activity in both FFI-OM samples, while PrP^Sc^ was never detected in any of the OM belonging to the other patients analyzed (Fig. 2B). The final RT-QuIC reaction products were collected (at 30 hours) digested with PK and analyzed by means of Western blotting. As previously observed^24^, seeds collected from OM of patients with FFI, sporadic or iatrogenic forms of CJD were able to produce final RT-QuIC aggregates partially resistant to PK digestion and characterized by a molecular weight ranging from 14 to 18 kDa (Fig. 3A). On the contrary, OM samples collected from patients with PD, AD or healthy subjects did not produce any PK resistant aggregate. Brain homogenate of patients with FFI and iCJD were used as positive controls.

### Estimation of PrP^Sc^ concentration in the olfactory mucosa by means of quantitative PMCA (qPMCA)

To estimate the concentration of PrP^Sc^ present in the olfactory mucosa of an FFI subject, we have performed a quantitative PMCA, as previously described^14^. First, we have carefully estimated the concentration of PrP^Sc^ present in a FFI brain homogenate used to calibrate the PMCA reaction. The estimation was carried out by comparing the PrP^Sc^ signal by Western blot with a known concentration of human recombinant PrP (Supplementary Fig. 2A). By this procedure we calculated that PrP^Sc^ concentration in the 10% brain homogenate of this patient was approximately 141 ng/ml. Thereafter, we have performed serial dilutions of this brain homogenate and used them for PrP^Sc^ detection after various rounds of PMCA. The results indicate that 1 round of PMCA enables to detect at around 10^−10^ to 10^−11^ g/ml of PrP^Sc^. Two or more rounds of PMCA allowed detection of 10^−13^–10^−14^ g/ml of PrP^Sc^ (Supplementary Fig. 2B). Olfactory mucosa of FFI patient resulted positive after 2 rounds of PMCA. According to our estimation the olfactory mucosa of this subject has a PrP^Sc^ concentration of 1.41 × 10^−14^ g/ml.

### Biochemical characterization of PMCA reaction products

The biochemical profile of the amplified PrP^Sc^ obtained from both FFI-OM samples was characterized by a predominance of the diglycosylated band of PrP with the unglycosylated one migrating at around 19 kDa^1^. As expected, PrP^Sc^ obtained from FFI brain homogenate showed a similar biochemical profile that did not change after amplification by PMCA (Fig. 3B)^26^. This pattern was then compared to that of PrP^Sc^ sCJD-type 1 or PrP^Sc^ sCJD-type 2 before and after PMCA. Although, sCJD brain homogenates showed their characteristic PrP^Sc^ biochemical profiles following PMCA the biochemical profile of both PrP^Sc^ changed and acquired a unique feature characterized by the prevalence of the diglycosylated band with the unglycosylated fragment migrating at around 19 kDa.

## Discussion

RT-QuIC and PMCA efficiently demonstrated the presence of PrP^Sc^ in urine^14^, CSF^19^, blood^15^ and olfactory mucosa^22^ of patients with prion diseases, at concentrations that are six orders of magnitude below the detection limits of the current diagnostic techniques, such as Western blot. RT-QuIC assay on OM samples obtained by nasal swabbing has been largely tested in patients with sporadic or genetic forms of CJD, showing a specificity of 100% and sensitivity of 97 and 75%, respectively, but never in patients with FFI or at risk of developing FFI.

In this work, we analyzed OM samples of 28 subjects 2 of whom carry the *D178N* mutation in the *PRNP* gene and already died with a clinical diagnosis of FFI. The other samples were collected from patients with different neurodegenerative disorders (including AD, PD and FTD) and healthy controls. RT-QuIC and PMCA analysis revealed the presence of PrP^Sc^ in both FFI-OM samples that were collected 4 and 10 months after the disease onset. In both cases, three rounds of amplification were enough to enable detection of PrP^Sc^. Moreover, through quantitative PMCA we have estimated that the OM sample analyzed contained about 1 × 10^−14^ g/ml of PrP^Sc^. This estimation should be carefully considered before being extended to other samples since it suffers from some limitations that might influence PMCA sensitivity. For instance, the efficiency of the nasal brushing itself and the dilution of samples before analysis might introduce technical variables to the assay. Even more important, the olfactory neurons are unique since they are replaced through life and the amount of PrP^Sc^ collected during each nasal brushing might also depend on this phenomenon^27^. PrP^Sc^ was not detected in patients affected by other neurodegenerative diseases. At this point, we wondered whether PMCA was able to faithfully amplify the PrP^Sc^ originally detected in the OM of FFI patients. To this aim, we have performed a biochemical characterization of the amplified products and, after PK digestion, we observed a PrP banding profile characterized by a predominance of the diglycosylated species with the unglycosylated one migrating at around 19 kDa. This profile was compared to that of a PrP^Sc^ collected from the brain homogenate of a patient who died for FFI^8^. Both profiles were similar and suggested that PMCA of OM samples might be able to maintain the biochemical feature of the PrP^Sc^ typically associated to FFI. To confirm our observation, we decided to perform PMCA analysis in FFI brain homogenates and in two cases of sporadic sCJD-type 1 or sCJD-type 2 with PrP^Sc^ biochemical profiles different to that of FFI. Following PMCA amplification, FFI-PrP^Sc^ did not change its biochemical profile, while PrP^Sc^ associated to each sCJD type converted to a PrP^Sc^ glycotype characterized by a predominance of the diglycosylated band with the unglycosylated one migrating at 19 kDa (type 2). Therefore, we have realized that, even though our PMCA conditions enable PrP^Sc^ amplification, the original biochemical features of the prion strain were not retained. Recent PMCA experiments^23^,^28^ revealed that the glycoform ratio or the electrophoretic mobility of different prion strains are not faithfully maintained after amplification using bank vole homogenates as substrate for the reaction. These results seem to be corroborated by *in vivo* experiments which demonstrated that intracerebral inoculation of different prion strain (including sCJD-type 1 and type 2) in bank voles^29^ or transgenic mice expressing the bank vole prion protein^25^ with methionine at codon 109 (Tg109M) gave rise to PrP^Sc^ with electrophoretic mobilities similar to that of the original inoculum but with a glycoform ratio characterized by a predominance of the diglycosylated PrP band^30^. All these results suggest that, although bank voles can amplify different strains of prion, they might amplify PrP^Sc^ with biochemical properties slightly different to that of the original prion strain. Certainly, additional factors other than PrP itself could play an important role in driving the fidelity of prion replication and further studies are necessary to clarify this intricate mechanism of amplification. PK digestion of RT-QuIC amplification products enabled detection of protease resistant recPrP useful for identifying reactions seeded with prion infected olfactory mucosa samples. However, differences in the Western blot banding profiles (between sCJD, iCJD or FFI) do not seem to have any correlation with distinct disease phenotypes.

Since the involvement of the olfactory system, including the olfactory bulb, in FFI has never been deeply investigated, we have hypothesized that PrP^Sc^ could have spread from the central nervous system (CNS) to the OM through axonal transport along nerve fibers, as it happens for the sporadic forms of CJD^31^. Otherwise, some mutations of the PrP^C^ gene, especially the D178N which is located in the hydrophobic core of the protein, are known to reduce the stability of the protein which is subsequently more prone to misfold^32^,^33^. This process might be favored in the nasal cavity, which is constantly exposed to chemical and physical stress^34^ which predisposes PrP^C^ to acquire less stable conformations, eventually leading to the formation of PrP^Sc^. If this were the case, the presence of PrP^Sc^ in the OM is not directly linked to disease progression but instead represents a completely independent event.

Our results demonstrated that the OM collected from symptomatic FFI patients contain minimal amount of PrP^Sc^. Therefore, considering the extremely high levels of sensitivity and specificity of PMCA and RT-QuIC, we are planning to investigate whether PrP^Sc^ might be detectable in subjects carrying the D178N mutation in the preclinical stage. There is only one study showing pre-symptomatic alterations in the thalamus of a D178N carrier who underwent to serial ^18^FDG-PET that was characterized by a selective hypometabolism in the thalamus 13 months before the clinical onset. No correlations between thalamic dysfunction and presence of PrP^Sc^ were analyzed^35^. Prospective longitudinal studies will enable to evaluate whether detection of PrP^Sc^ in OM collected from subjects of a well characterized Italian FFI family, which is currently involved in a preventive clinical trial with doxycycline, might be used as quantifiable biomarker to monitor the effect of the drug on PrP replication and disease progression^33^.

## Materials and Methods

### Ethical approval

The study was approved by the institutional review board of Carlo Besta Neurological Institute and performed according to the guidelines approved by the ethics committee. Written informed consent for participation in research was done in accordance with the Declaration of Helsinki (1964–2008) and the Additional Protocol on the Convention of Human Rights and Biomedicine concerning Biomedical Research (2005).

### Olfactory mucosa brushing procedure

A step-by-step tutorial video of the nasal brushing procedure is available at: https://www.youtube.com/watch?v=wYb9W3u6uMY. This procedure was performed using flocked swabs (FLOQSwabs^TM^ Copan Italia, Brescia, Italy), as previously described^22^,^36^.

### Brain homogenates preparation

Frontal cortex of patients with Fatal Familial Insomnia (FFI-D178N), Alzheimer’s disease (AD), Frontotemporal Dementia (FTD) sporadic Creutzfeldt-Jakob disease (sCJD) and iatrogenic Creutzfeldt-Jakob disease (iCJD) were homogenized in phosphate buffer (pH 7.4, Sigma) at 10% (weight/volume). Samples were centrifuged (Eppendorf Centrifuge) at 4 °C, 800 × g, 1 minute in order to remove cellular debris. Supernatant was collected and stored at −80 °C for further use.

### Olfactory mucosa cells collections

Olfactory mucosa (OM) cells were collected from the mid-part of the inferior turbinate by brushing with a cotton swab, after treating the nasal mucosa of the patients with a topical anesthetic. Cotton swab was subsequently immersed in saline solution and olfactory cells collected from the brush by means of vortexing.

### Olfactory mucosa cells preparation for PMCA analysis

Olfactory mucosa cells were pelleted for 20 minutes at 800 × g at 4 °C. The supernatant was removed and approximately 2 μl of the pellet was collected and transferred into a tube containing 25 μl of PBS. The latter was sonicated (approximately 280 W) with a Microsonicator QSonica (Q700) until the pellet was dispersed and 10 μL of this solution was added to 90 μL of specific PMCA substrate and subjected to several rounds of amplification.

### Substrate preparation for PMCA analysis

Bank voles carrying the M109 PrP genotype were sacrificed with carbon dioxide and immediately perfused using phosphate-buffered saline (PBS) plus 5 mM ethylendiaminetetraacetic acid (EDTA, Sigma). Brains were collected and homogenized in conversion buffer (PBS, 150 mM NaCl, 1% Triton X-100) at 10% (weight/volume). Heparin (100 μg/mL, Sigma), digitonin (0.05%, Sigma) and 3 teflon beads were added to the homogenates to increase PMCA efficiency. To avoid contamination, perfusion and brain homogenates were carried out in a prion-free laboratory.

### PMCA procedure

PMCA was performed as previously reported^36^. Briefly, 10 μL of sonicated olfactory mucosa samples was added to 90 μL of fresh substrate and subjected to 96 cycles of PMCA with the use of a microsonicator (model Q700, Qsonica). Each cycle consisted of 29 minutes 30 seconds of incubation at 37–40 °C, followed by a 30-second pulse of sonication set at a potency of 260–280 W. After one round of PMCA, an aliquot of the amplified material was diluted by a factor of 10 into fresh brain homogenate and an additional round was performed. Each sample was subjected to 7 rounds of PMCA. Considering our ultrasensitive level of amplification, all PMCA reactions were prepared under rigorous prion-free conditions. Overall, in order to avoid any contamination, appropriate number of negative controls were added in each PMCA assay. No contamination or *de novo* generation of prions were observed.

### SDS-PAGE and Western blotting

Ten microliters of final PMCA products was treated with 50 μg/mL of PK (Invitrogen) for 1 hour at 37 °C under shaking (550 rpm). Digestion was stopped by the addition of LDS-PAGE loading buffer (Life Technologies), samples were heated at 100 °C for 10 minutes and loaded into 12% Bolt Bis-Tris Plus gels (Life Technologies). Proteins were separated by means of SDS-PAGE, transferred onto Polyvinylidene difluoride (PVDF, Millipore) membrane and incubated with 5% (wt/vol) dry nonfat milk in 0.05% (vol/vol) Tween-20 (prepared in Tris-HCl) for 1 h at room temperature with shaking. PVDF membranes were finally incubated with anti-PrP antibody 6D11 (epitopes 93–109, Covance) and developed with chemiluminescent system (ECL Prime, GE Healthcare Amersham).

Ten microliters of RT-QuIC reaction products was digested with 50 μg/mL of PK at 37 °C for 30 minutes under shaking (550 rpm). Digestion was stopped by the addition of LDS-PAGE loading buffer, samples were heated at 100 °C for 10 minutes and loaded into 17% Bolt Bis-Tris Plus gels. Proteins were separated by means of SDS-PAGE, transferred onto Polyvinylidene difluoride (PVDF) membrane and incubated with 5% (wt/vol) dry nonfat milk in 0.05% (vol/vol) Tween-20 (prepared in Tris-HCl) for 1 h at room temperature with shaking. PVDF membranes were finally incubated with anti-PrP antibody SAF-84 (epitopes 160–170, Spi Bio) and developed with chemiluminescent system (ECL Prime).

### Recombinant PrP production for RT-QuIC analysis

Truncated Bank voles PrP (BvPrP(90-231)) construct was purchased from GenScript. The construct was expressed in *Escherichia coli* BL21 (DE3) cells (Stratagene). Freshly transformed overnight culture was inoculated into Luria Bertani (LB) medium and 100 μg/mL ampicillin. At 0.8 OD600 expression was induced with isopropyl b-D galactopyranoside (IPTG) to a final concentration of 0.75 mM. Cells were grown in a BioStat-B plus fermentor (Sartorius). The cells were lysed by a homogenizer (PandaPLUS 2000) and the inclusion bodies were suspended in buffer containing 25 mM Tris-HCl, 5 mM EDTA, 0.8% TritonX100, pH 8, and then in bi-distilled water several times. Inclusion bodies containing BvPrP(90-231) were dissolved in 5 volumes of 8 M guanidine hydrochloride (GndHCl), loaded onto pre-equilibrated HiLoad 26/60 Superdex 200-pg column, and eluted in 25 mM Tris–HCl (pH 8), 5 mM ethylenediaminetetraacetic acid (EDTA), and 6 M GndHCl at a flow/rate of 2 mL/min. Proteins refolding was performed by dialysis against refolding buffer (20 mM sodium acetate and 0.005% NaN_3_ (pH 5.5)) using a Spectrapor membrane. Purified protein was analyzed by SDS-polyacrylamide gel electrophoresis under reducing conditions and Western blot.

### Olfactory mucosa cells preparation for RT-QuIC assay

Olfactory cells were collected from the brush by means of vortexing. The brush was then removed and the cells were pelleted for 20 minutes at 800 × g at 4 °C. The supernatant was removed, and approximately 2 μl of the pellet was collected and transferred into a tube containing 25 μl of PBS. The latter was sonicated (approximately 280 W) until the pellet was dispersed and 2 μL of this solution was further diluted in 18 μL of freshly prepared PBS. Two μL of the final solution was added to each reaction well.

### Real-Time Quaking Induced Conversion procedure

After purification, aliquots of the recombinant BvPrP(90-231) were stored at −80 °C in 10 mM phosphate buffer (pH 5.8). Before each test, the protein solution was allowed to thaw at room temperature and filtered through a 100 kDa Nanosep centrifugal device (Pall Corporation). The concentration of recPrP was determined by measuring the adsorbance at 280 nm. Samples were blindly analyzed at least three times (by different operators) in triplicate in a black 96-well optical flat bottom plate (Thermoscientific) and results showed a high degree of consistency.

The final reaction volume was 100 μL and the reagents (Sigma) were concentrated as follow: 150 mM NaCl, 0.002% SDS, 1X PBS, 1 mM EDTA, 10 μM ThT and 0.1 mg recPrP ml^−1^. To avoid contamination, reaction mix was prepared in a prion-free laboratory. After the addition of 2 μL of olfactory mucosa samples, the plate was sealed with a sealing film (Thermoscientific) and inserted into a FLUOstar OPTIMA microplate reader (BMG Labtech). The plate was shaken for 1 minute at 600 rpm (double orbital) and incubated for 1 minute at 55 °C. Fluorescence reading (480 nm) were taken every 15 minutes (30 flashes per well at 450 nm). Given the rapid response, a specific threshold was set to decrease the likelihood of false positives. A sample was considered positive if the mean of the highest two fluorescence values (AU) of the replicates was higher than 10.000 AU and at least two out of three replicates crossed the threshold that was set at 30 hours. This reaction cutoff was established because in all the experiments that were seeded with the brain homogenates of patients with Alzheimer’s disease, no positive RT-QuIC reactions were observed until after 30 hours. In rare occasions, we could observe the aggregation of 1 replicate out of 3 before the threshold. In these cases, we have performed additional analysis of the samples in quadruplicate before considering it negative. The sample was confirmed negative if none or only one replicate (out of four) crossed the threshold before 30 hours. This allowed us to maximize the accuracy of the analysis thus reducing the risk of false positive or false negative results.

## Additional Information

**How to cite this article:** Redaelli, V. *et al*. Detection of prion seeding activity in the olfactory mucosa of patients with Fatal Familial Insomnia. *Sci. Rep.* **7**, 46269; doi: 10.1038/srep46269 (2017).

**Publisher's note:** Springer Nature remains neutral with regard to jurisdictional claims in published maps and institutional affiliations.

## Supplementary Material

Supplementary Information
