# Supplementary material for: Detection of prion seeding activity in the olfactory mucosa of patients with Fatal Familial Insomnia
Source: Sci Rep. 2017 Apr 7;7:46269. doi: 10.1038/srep46269 (PMC5384244; doi:10.1038/srep46269)
Supplement: Supplementary Information [file srep46269-s1.pdf]

Detection of prion seeding activity in the olfactory mucosa of patients with Fatal Familial Insomnia

Veronica Redaelli, Edoardo Bistaffa, Gianluigi Zanusso, Giulia Salzano, Luca Sacchetto, Martina Rossi, Chiara Maria Giulia De Luca, Michele Di Bari, Sara Maria Portaleone, Umberto Agrimi, Giuseppe Legname, Ignazio Roiter, Gianluigi Forloni, Fabrizio Tagliavini, Fabio Moda

SUPPLEMENTARY MATERIALS

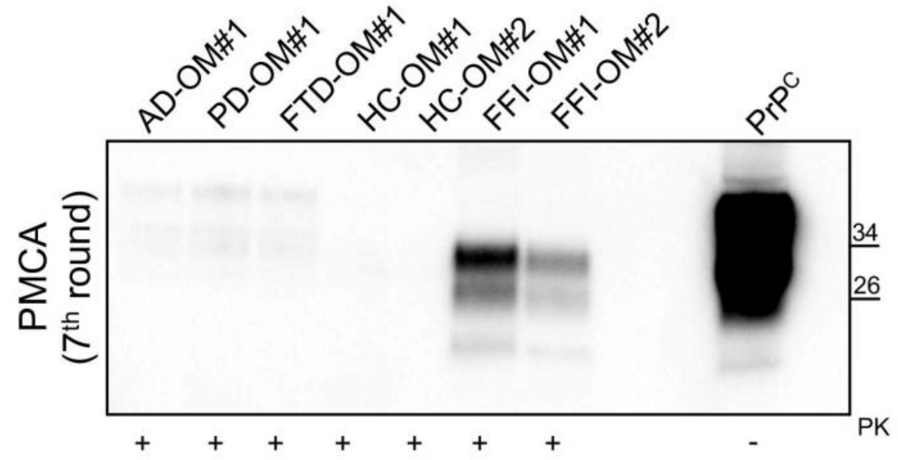

Supplementary Figure 1. PMCA analysis of OM samples from patients with other neurodegenerative disorders. PMCA analysis of OM samples collected from 2 FFI subjects, 3 patients with other neurodegenerative diseases, including AD (n=1), PD (n=1) and FTD (n=1) and 2 healthy controls. Seven rounds of amplification were performed. The signal of PrP^{Sc} was assessed by means of Western blotting, after PK digestion, with the 6D11 antibody. Numbers in the right of the blots indicate the position of molecular weight marker.

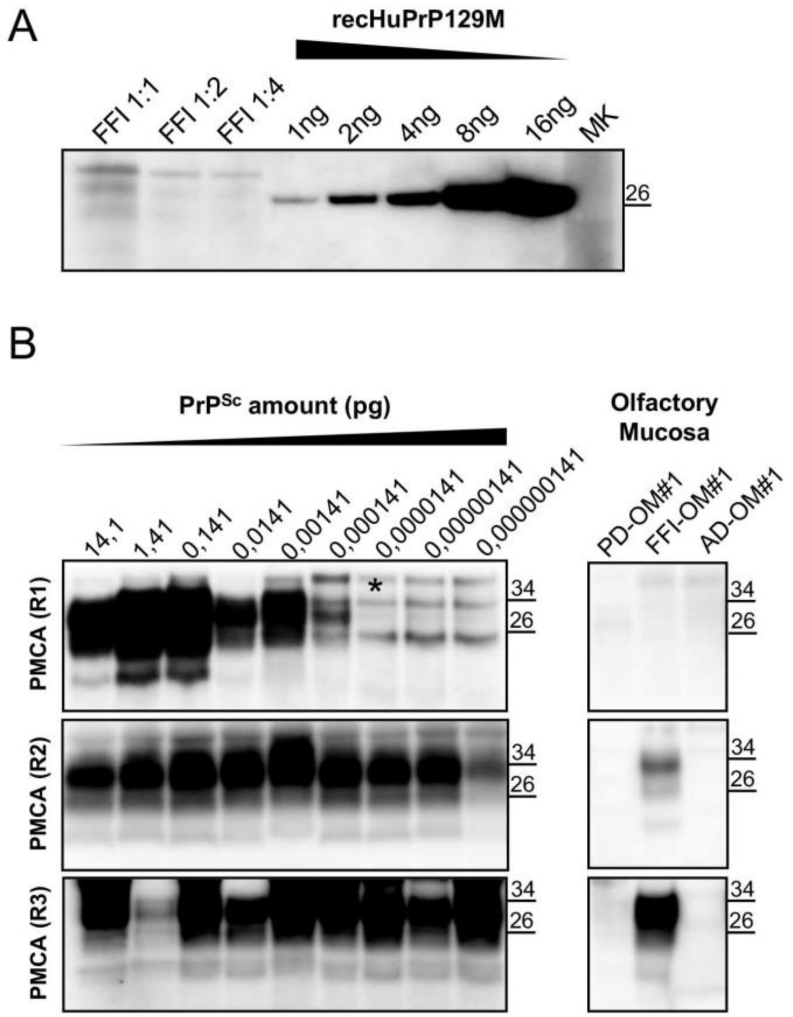

Supplementary Figure 2. Quantitative PMCA (qPMCA) for estimating PrP^{Sc} concentration in OM samples of FFI patients. (**A**) The intensity of the PrP^{Sc} signal obtained after PK digestion from different dilutions of FFI brain homogenate (20-fold concentrated) was compared to that of known concentrations of recombinant human prion protein with methionine at codon 129 (recHuPrP129M). Mw refers to molecular weight marker. (**B**) Quantified FFI-PrP^{Sc} was spiked in bank vole brain homogenate and PMCA experiments were performed along with FFI-OM#1 sample. OM collected from patients with PD and AD were used as negative controls. The signal of PrP^{Sc} was assessed by means of Western blotting, after PK digestion, with the 6D11 antibody. Asterisk indicates unspecific signal. Numbers in the right of the blots indicate the position of molecular weight marker.

A (Figure 2A. Before PMCA)

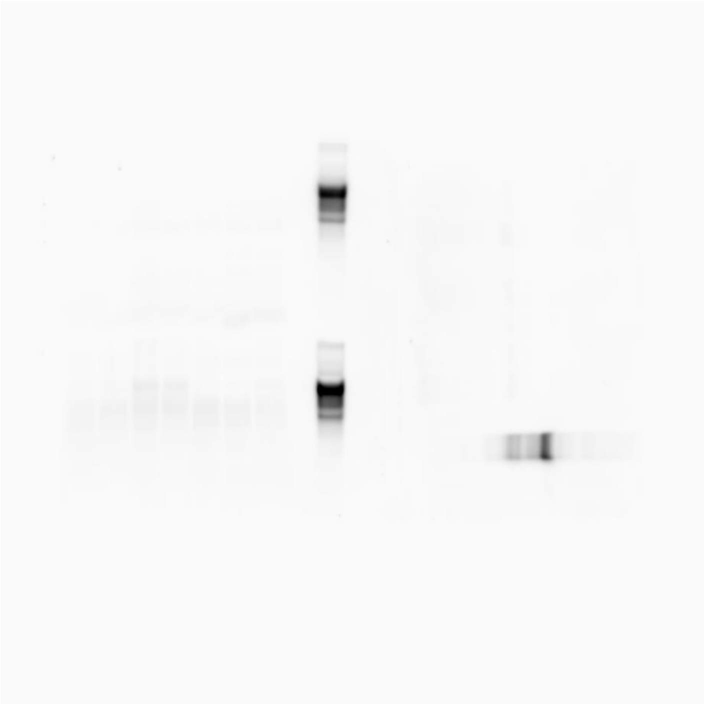

B (Figure 2A. After PMCA 3rd round)

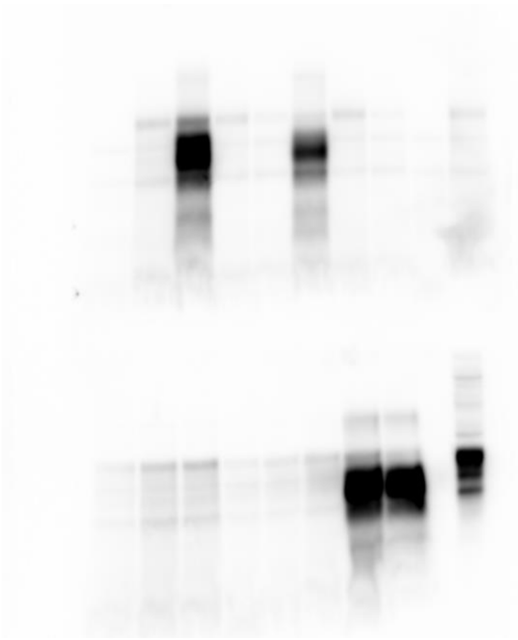

Raw pictures. (**A**) Raw gels used to prepare Figure 2A “Before PMCA”. The picture on top/left and the picture on the right were used. (**B**) Raw gels used to prepare Figure 2A “After PMCA 3rd round”. Both pictures were used.
